# Sublethal and transgenerational effects of synthetic insecticides on the biological parameters and functional response of *Coccinella septempunctata* (Coleoptera: Coccinellidae) under laboratory conditions

**DOI:** 10.3389/fphys.2023.1088712

**Published:** 2023-01-16

**Authors:** Rahat Afza, Ayesha Afzal, Muhammad Asam Riaz, Muhammad Zeeshan Majeed, Atif Idrees, Ziyad Abdul Qadir, Muhammad Afzal, Babar Hassan, Jun Li

**Affiliations:** ^1^ Department of Entomology, College of Agriculture, University of Sargodha, Sargodha, Pakistan; ^2^ Guangdong Key Laboratory of Animal Conservation and Resource Utilization, Guangdong Public Laboratory of Wild Animal Conservation and Utilization, Institute of Zoology, Guangdong Academy of Sciences, Guangzhou, China; ^3^ Institute of Molecular Biology and Biotechnology, The University of Lahore, 1-Km Defense Road, Lahore, Pakistan; ^4^ Guizhou Provincial Key Laboratory for Agricultural Pest Management, Institute of Entomology, Ministry of Agriculture, Guizhou University, Guiyang, China; ^5^ Honeybee Research Institute, National Agricultural Research Centre, Park Road, Islamabad, Pakistan; ^6^ Department of Entomology and Wildlife Ecology, University of Delaware, Newark, DE, United States; ^7^ University of the Sunshine Coast, Maroochydore, QLD, Australia

**Keywords:** coccinellid beetles, seven-spotted lady beetle, synthetic insecticides, sublethal exposure, life parameters, functional response

## Abstract

Synthetic insecticides have been an inevitable part of plant protection throughout the world. Sublethal effects of these chemicals on beneficial insect species are one of the contemporary issues these days. Using the age-stage, two-sex life table model, this study evaluated the sublethal and transgenerational effects of six synthetic insecticides (imidacloprid, thiamethoxam, lambda-cyhalothrin, cypermethrin, chlorpyrifos and profenofos) commonly applied to winter vegetables, on the fitness and predation of the seven-spotted ladybeetle, *Coccinella septempunctata*, which is an efficient predator of aphids worldwide. According to results, all insecticides at their sublethal doses (LC_30_) significantly suppressed the emergence of adults, adult weight, fertility and fecundity of the parental generation compared to control treatment. The larval stage was prolonged and oviposition, fecundity and total longevity of the adult beetles were decreased in unexposed progeny whose parents were exposed to sublethal doses of all insecticides. Moreover, the biological parameters of adults, including the intrinsic rate of increase (*r*), finite rate of increase (*λ*) and net reproductive rate (*R*_0_) were significantly reduced when exposed to sublethal doses of insecticides. The predation rate of the F_1_ generation adults was also decreased after exposure to the sublethal doses of insecticides. However, chlorpyrifos, profenofos, lambda-cyhalothrin and cypermethrin exhibited more deleterious effects on the fitness and population parameters of beetles than imidacloprid and thiamethoxam.

## 1 Introduction

The ladybeetle, *Coccinella septempunctata* L. (Coleoptera: Coccinellidae) is a well-known predator of various species of agricultural insect pests worldwide including Pakistan (Hodek and Michaud 2013; Saljoqi and Ali 2013). It is a generalist predator often observed on arable crops, feeding on aphids, whiteflies, psyllids, mites, and scales that are destructive insect pests of several crops (Bianchi and Van der Werf 2003; Farooq and Tasawar 2008; Sarwar 2013; Afza et al., 2019; He et al., 2019; Jiang et al., 2019; Afza et al., 2021). This species is considered a useful biological control agent of brassica aphids because of its voracious appetite. It feeds on more than twelve aphid species and rapidly responds to aphid populations (Jiang et al., 2019).

The use of synthetic insecticides by farmers is the predominant practice to keep pest populations below economic injury level (Afza et al., 2019; Afza et al., 2021). Chemical insecticides are cost-effective, easy to use and adequate against target pests. However, their excessive usage may have various adverse effects on beneficial arthropods in agroecosystems. The predatory potential of *C. septempunctata* is negatively affected after feeding on contaminated prey and plant material during foraging. Both direct contact with spray droplets and residues of insecticides affect its predation (Desneux et al., 2007; Afza et al., 2019; Afza et al., 2021). The deleterious effects of insecticides on coccinellid beetles include acute toxicity and changes in physiological, biochemical and behavioural processes. A lethal dose may not represent the overall adverse effects of synthetic insecticides, as sublethal concentrations do also have effect on insect behaviour and physiology (Galvan et al., 2005; Desneux et al., 2007; Rahmani and Bandani 2016). Effects of sublethal doses on the physiology of insects range from immunological to biochemistry, neurophysiological, sex ratio, fecundity, longevity and weight. Behavioural effects include disturbed mobility, orientation, feeding behaviour and learning performance (Galvan et al., 2005; Desneux et al., 2007; Afza et al., 2021). Sublethal doses of synthetic insecticides also have deleterious effects on the functional response of predatory beetles and other biological control agents (He et al., 2012; Jiang et al., 2019).

Indigenous farmers in Pakistan use several insecticides to manage aphids and other pests on their crops. All these insecticides have different ranges of toxicity and adverse effects on *C. septempunctata* (Arshad et al., 2017; Afza et al., 2019; Afza et al., 2021). Although biological control is an integral part of integrated pest management (IPM) programs, chemical control is the primary and most effective method for combating pests. However, some of the synthetic insecticides are relatively safer to be used as components of IPM programs. Therefore, knowledge of the non-target effects of insecticides on the behavioural traits, growth and development of biological control agents, is essential for an effective incorporation of insecticides in successful pest management programs (Tillman and Mulrooney 2000; Youn et al., 2003; Galvan et al., 2005; Bozsik 2006; Stark et al., 2007; Tengfei et al., 2019).

Several studies have reported on the lethal and sublethal effects of pesticides on predatory coccinellid beetles, showing that pesticides are harmful to these natural enemies (Urbaneja et al., 2008; Cabral et al., 2011; He et al., 2012; He et al., 2019; Jiang et al., 2019). However, data on age-related life table analyses and demographic parameters of *C. septempunctata* to evaluate the effects of currently used insecticides is still lacking. No study has evaluated the sublethal impacts of formulated insecticides commonly used by farmers for the management of aphids in Pakistan. Hence, an overall risk assessment of the exposure of *C. septempunctata* to currently-used synthetic insecticides is necessary. Apropos of the context, this study was aimed to assess the sublethal effects of six commonly-used synesthetic insecticides at their lower concentrations (LC_30_), on the fecundity, development and demographic parameters of *C. septempunctata* based on the life table theory. Moreover, the effects of their sublethal doses on the functional response of *C. septempunctata* were also assessed. The results of the current study can be useful for guiding assessment of the compatibility between the *C. septempunctata* and different insecticides in future IPM strategies for aphids. They would also contribute to the conservation of *C. septempunctata* in the field.

## 2 Materials and methods

### 2.1 Insect rearing

*C. septempunctata* adults were collected from canola (*Brassica napus* L.) fields managed without application of insecticides, at a farm area of the University of Sargodha, Pakistan (32°4′ N; 72°40′ E). Adult beetles were identified using identification keys described by (Poorani 2002; Rafi et al., 2005; Saeed et al., 2016) and were sorted into fifty couples (male and female). Then each pair of male and female coccinellid beetles was released into glass bowl (7.0 × 2.5 cm), which were then covered with muslin cloth and placed in an incubator (at 25 ± 2°C and 60 ± 5% R.H.), to obtain eggs for single cohort progeny. Collected adults and newly-hatched larvae of beetles were offered twigs and branches of canola infested by aphids (Brevicoryne brassicae L.) in a bowl. Aphids collected from the canola field were provided after every 12 h. For this purpose, the canola crop was sown in the campus and no insecticide was sprayed on the crop. Adult beetles were also offered a continuous supply of 10% glucose solution *via* a soaked cotton wool plug (Sarwar and Saqib 2010; Afza et al., 2019; Afza et al., 2021).

### 2.2 Selection and source of insecticides

A field survey was conducted in the brassica growing region of Punjab (Pakistan) for 2 years. One hundred farmers were asked about the insecticides sprayed for the control of aphids on brassica and other winter crops every year. Survey results revealed six insecticides which were commonly sprayed by the farmers to manage their crop pests. These included imidacloprid, thiamethoxam, lambda-cyhalothrin, cypermethrin, chlorpyrifos and profenofos. Hence, these six insecticides were selected for the current study. Trade names, type of formulations, percent active ingredients (a.i.) and sources of the tested insecticides are given in Supplementary Table S1.

### 2.3 Determination of sublethal concentration (LC_30_) values

The experiment for the determination of lethal and sublethal concentration values was conducted following Afza et al. (2021). Based on mortality results obtained from preliminary assays (0–90%), five to six concentrations of each insecticide were prepared by dissolving them in water. A sample of 25–30 fourth instar larvae of *C. septempunctata* were treated with the concentrations of each insecticide separately. The control involved treatment of larvae with water. The experiment was replicated five times. Larvae were placed on ice to reduce their mobility during the bioassays. Then, 1 μl of each insecticide at different concentration and water (for control) was applied separately on the mesonotum of fourth instar larvae using a micropipette.

Treated larvae were placed in Petri-plates (90 mm diameter) lined with moistened Whatman No. 1 filter paper discs. To prevent the larvae from escaping, the plates were covered with PVC film which had small holes for aeration and were placed in an incubator (at 25 ± 2°C and 60 ± 5% R.H). Aphids were provided *ad-libitum* daily. Larval mortality was recorded 24 h after treatment. All beetles used in this experiment were 12–15 weeks old. Probit analysis using the Polo Plus^®^ software was performed to calculate the median lethal concentrations (LC_50_ and LC_30_) values for each insecticide. The toxicity of sublethal dose (LC_30_) was confirmed in a separate test against the fourth instar larval beetle.

### 2.4 Effects of LC_30_s on parental generation (F0) of *C. septempunctata*

Three groups of freshly laid (0–6 h old) 100 eggs were placed on moistened Whatman No. 1 filter paper discs lined in Petri-plates (90 mm diameter). After inspection every 6 h, newly-hatched larvae were transferred to new Petri-plates and were fed on mixed instars of canola aphids (*Brevicoryne brassicae* L). All Petri-plates were incubated in an environmental chamber at 25 ± 2°C, 60 ± 5% R.H. and 14:10 h (L: D) photoperiod. When larvae reached the fourth instar (0–12 h old), sublethal concentrations (LC_30_) of each insecticide was applied topically. The treatment method was similar as described above. Forty-five larvae (15 larvae per replicate) were treated in each treatment and the experiment was repeated thrice. For the control treatment, larvae were treated with water. Fourth instar larvae were chosen for this experiment because of their lower natural mortality, compared to that of the initial three instars. Larval mortality, percentage of pupae formed and adult emergence from pupae were recorded at every 12 h until the adult stage. Larvae found motionless were considered dead. Newly-emerged adults were weighed and fed with B. brassicae individuals and 10% glucose solution. Male and female beetles from the same treatment were paired the same or second day of emergence and were observed daily to record their survival rate, fecundity and egg hatchability, until all the individuals were dead. For the fecundity, fifteen couples were tested per insecticide treatment.

### 2.5 Effects of LC_30_s on demographic growth parameters of F1 generation

A total 45–50 eggs (0–6 h old) from three to four couples of the parental generation, from the same treatment, were separated and kept in Petri-plates. Eggs were checked at 12 h intervals and newly-emerged larvae were transferred to new Petri-plates and fed on aphids. Each larva was considered as one replicate and its developmental time was monitored until pupation. Newly-emerged adults were sexed (12–15 couples) and their fecundity, survival rate and developmental time were recorded until the death of all individuals of the F_1_ generation.

### 2.6 Effects of LC_30_s on functional response of F1 generation

F1 adult beetles starved for 24 h, were confined on canola foliage in Petri-plates (150 mm diameter) lined with moistened Whatman No. 1 filter paper discs and were offered B. brassicae aphid individuals at five densities, i.e. 20, 80, 160, 320 and 640 aphid individuals, as a food source. These aphids were pre-exposed to sublethal doses (LC_30_) of the insecticides. Water was used as control treatment. Twelve replications were carried out for each aphid density treatment. Individual coccinellids were offered insecticide-treated aphids. The data on aphid consumption by each beetle was recorded. One replicate under each density served as no-predator control, to determine the mortality of aphids after treating with insecticides. Experiments were conducted under controlled condition at 25 ± 2°C, 60 ± 5% R.H, and 14:10 h (L: D) photoperiod.

### 2.7 Statistical analysis

The data obtained from the toxicity bioassays were corrected using Abbott’s formula before analysis. The LC_50_ and LC_30_ values were calculated for each insecticide by probit analysis using the Polo Plus^®^ software (Finney 1971). The normality of the parental generation (F_0_) data was assessed using the Kolmogorov-Smirnov test in SPSS^®^ version 21.0 (SPSS Inc, Chicago, IL, United States) and data were analysed using one-way ANOVA in GraphPad Prism version 7.0 (GraphPad Software Inc, San Diego, CA, United States). Treatment means were compared by Tukey’s honest significant difference (HSD) *post hoc* test. Life table data were analysed using age-stage, two-sex life table using Two Sex-MS Chart program (Chi 1988). Standard errors of the life table parameters were calculated *via* 100,000 bootstrap replicates to obtain stable S.E. estimates. Sigma Plot^®^ version 12.0 (Systat Software, Inc, San Jose, CA, United States) was used to generate curves for all population parameters including age-stage specific survival rate (Sxj), age-stage life expectancy (exj), age-specific survival rate (lx), age-specific fecundity (mx), net maternity (lx*mx) and reproductive value (vxj). Moreover, a two-step approach recommended by Juliano (2001) was used to determine the type of functional response and its parameters. Further details are given in Supplementary Table S1 and Supplementary Table S2.

## 3 Results

### 3.1 Toxicity bioassay

The LC_50_ values of the six insecticides against *C. septempunctata* larvae were 256.6, 191.3, 122.3, 92.2, 153.1 and 235.9 mg a. i./ml for imidacloprid, thiamethoxam, profenofos, chlorpyrifos, lambda-cyhalothrin and cypermethrin, respectively. The highest LC_50_ was recorded for imidacloprid, while the minimum LC_50_ was observed in chlorpyrifos. The organophosphate insecticides, profenofos and chlorpyrifos, exhibited comparatively lower LC_50_ values against the fourth instar larvae of *C. septempunctata* than that of the other insecticides. The LC_30_ values of these insecticides were determined as 158.7, 117.5, 75.5, 56.4, 95.9, and 110.4 mg a. i./ml for imidacloprid, thiamethoxam, profenofos, chlorpyrifos, lambda-cyhalothrin and cypermethrin, respectively.

### 3.2 Effects of LC_30_s on parental (F0) *C. septempunctata* generation

**FIGURE 1:**
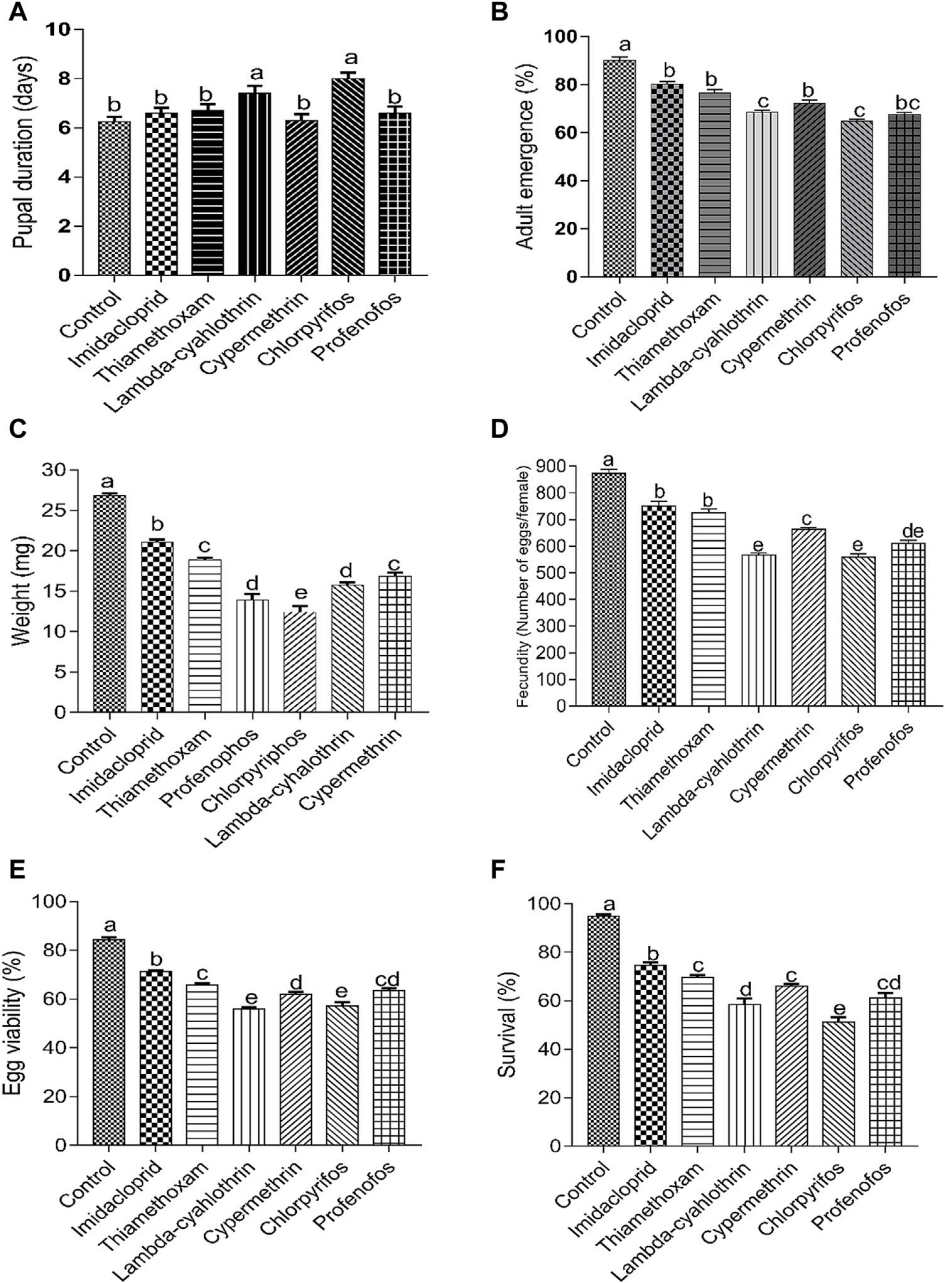
Effect of sublethal concentrations (LC_30_) of synthetic insecticides on the pupal duration **(A)**, adult emergence **(B)**, weight of newly-emerged adults **(C)**, fecundity (eggs/female) **(D)** egg viability **(E)** and adult survival **(F)** of the F_0_ generation of *Coccinella septempunctata*. Larvae of *Coccinella septempunctata* were exposed to sublethal concentrations of imidacloprid, thiamethoxam, profenofos, chlorpyrifos, lambda-cyhalothrin and cypermethrin. Treatment means were compared by Tukey’s Honest Significant Difference (HSD) *post hoc* test. Means with the same letters are not significantly different at *p* ≤ 0.005.

The treatment of larvae with sublethal concentrations of the six insecticides had significant negative effects on the biological parameters of *C. septempunctata* compared to the control groups (Figure 1). Exposure to sublethal concentration of lambda-cyhalothrin, chlorpyrifos and profenofos, significantly increased the pupal duration of beetles (>7 days), compared to the other insecticides and control (water-treated) insects (<6.8 days) (F = 6.22; *p* < 0.0001) (Figure 1A). The adult emergence rate of insecticide-treated *C. septempunctata* was also significantly affected compared to the control insects (F = 58.84; *p* < 0.0001). The sublethal doses of lambda-cyhalothrin, chlorpyrifos and profenofos significantly reduced the emergence rate compared to insects treated to cypermethrin, imidacloprid and thiamethoxam. A higher adult emergence rate was recorded for control (water treated) insects (Figure 1B). Similarly, the weight of newly-emerged beetles treated with insecticides was reduced compared to control groups (F = 5.09; *p* = 0.001). A significantly higher decrease in weight of adult beetles was observed after treatment with chlorpyrifos, followed by lambda-cyhalothrin and profenofos. Thiamethoxam and cypermethrin caused similar effects on the weight of beetles (Figure 1C). The fecundity rate of females was also significantly suppressed after treatment with sublethal concentrations of the six insecticides, compared to control treatment (F = 83.01; *p* < 0.0001). The most significant reduction in fecundity occurred after treatment with lambda-cyhalothrin, chlorpyrifos and profenofos followed by cypermethrin. Thiamethoxam and imidacloprid caused similar effects on the fecundity of beetles. A higher fecundity was observed in beetles treated with water (872.4 ± 15.75 eggs) (Figure 1D). Egg hatchability (%) (F = 103.40; *p* < 0.0001) and survival (%) (F = 80.50; *p* < 0.0001) of *C. septempunctata* treated with sublethal doses of six insecticides was also significantly reduced compared to control groups. A sublethal treatment of chlorpyrifos and lambda-cyhalothrin, significantly reduced the egg viability and survival of beetles compared to the rest of insecticides. However, egg hatchability and survival of water-treated beetles were substantially higher compared to insecticides-treated beetles (Figure 1E, F).

### 3.3 Effect of LC_30_s on the demographic growth parameters of *C. septempunctata*

Results of the effect of sublethal doses of the insecticides on the developmental time of the F_1_ generation are presented in Table 1. All sublethal insecticidal treatments did not significantly affect the egg hatching and first instar larval period of the beetles (egg hatching duration: F = 4.55; *p* = 0.090; first instar duration: F = 12.33; *p* = 0.10). The larval duration of the second instar of all insecticide-treated beetles significantly lengthened, compared to control treatment (F = 47.15; *p* = 0.040). Here, lambda-cyhalothrin had the most significant effect on this duration compared to other insecticidal treatments. Similarly, the period of the third instar larvae was significantly increased after treatment with lambda-cyhalothrin, chlorpyrifos, and profenofos compared to other insecticides. Overall, the third instar larval period of insecticide-treated beetles was substantially longer compared to that of water-treated beetles (F = 43.65; *p* = 0.030). The fourth instar period of beetles treated with lambda-cyhalothrin and cypermethrin was significantly increased compared to thiamethoxam and imidacloprid (F = 25.19; *p* = 0.032). Moreover, the pupal duration of beetles treated with profenofos significantly lengthened compared to water-treated beetles. Nevertheless, the pupal period of beetles treated with the other insecticides did not differ significantly from control treatment and with profenofos (F = 51.23; *p* = 0.041). Adult duration and total longevity of insecticide-treated beetles were reduced considerably compared to water-treated beetles (Adult duration: F = 157.11; *p* = 0.002; overall longevity: F = 412.13; *p* = 0.001) **(** Table 1).

**TABLE 1:** Means (days ±SE) developmental time of the different life stages of *Coccinella septempunctata* F1 generation.

Basic statistic	Control	Imidacloprid	Thiamethoxam	Lambda-cyhalothrin	Cypermethrin	Chlorpyrifos	Profenofos
Egg duration	3.20 ± 0.10a	3.20 ± 0.1a	3.20 ± 0.10a	3.79 ± 0.1a	3.29 ± 0.1a	3.29 ± 0.1a	3.29 ± 0.10a
Larvae 1 duration (d)	3.28 ± 0.09a	3.27 ± 0.09a	3.27 ± 0.09a	3.26 ± 0.09a	3.26 ± 0.09a	3.26 ± 0.09a	3.26 ± 0.09a
Larvae 2 duration (d)	3.30 ± 0.13c	3.39 ± 0.10b	3.40 ± 0.10b	3.55 ± 0.12a	3.48 ± 0.11b	3.45 ± 0.10b	3.45 ± 0.10b
Larvae 3 duration (d)	3.59 ± 0.15c	3.70 ± 0.16b	3.73 ± 0.15b	3.82 ± 0.17a	3.68 ± 0.19b	3.85 ± 0.17a	3.85 ± 0.14a
Larvae 4 duration (d)	4.0 ± 0.11c	4.17 ± 0.11b	4.18 ± 0.12b	4.27 ± 0.17a	4.27 ± 0.17a	4.20 ± 0.15ab	4.20 ± 0.15ab
Pupae duration (d)	6.36 ± 0.41b	6.42 ± 0.12ab	6.43 ± 0.12ab	6.63 ± 0.23ab	6.54 ± 0.22ab	6.63 ± 0.23ab	7.04 ± 0.21a
Adult duration (d)	55.48 ± 0.63a	53.98 ± 2.13b	52.22 ± 0.65b	45.58 ± 2.32d	46.25 ± 2.45c	47.96 ± 2.13c	49.88 ± 2.09bc
Total longevity (d)	67.77 ± 4.91a	64.8 ± 4.73ab	52.33 ± 4.76b	57.81 ± 4.58c	58.01 ± 4.67c	59.25 ± 4.80bc	61.04 ± 4.94bc

Means sharing same letters in rows are not significantly different from one another at *p* > 0.05.

Results of the effects of the six insecticides at LC_30_, on the population parameters of the F_1_ generation of *C. septempunctata* are presented in Table 2. Sublethal doses of all six insecticides significantly reduced the intrinsic rate of increase (*r*) and finite rate (λ) of the beetles. However, the finite rate of beetles treated with thiamethoxam and imidacloprid was similar to that of water-treated beetles. Sublethal treatments of chlorpyrifos, profenofos, and lambda-cyhalothrin significantly decreased the intrinsic rate of increase compared to the other three insecticides (*p < 0.05*). All insecticides at sublethal doses (LC_30_) significantly reduced the net reproduction rate (R0) (F = 351.65; *p* < 0.0001) and increased the mean generation time (*T*) (F = 253.15; *p* = 0.002) compared to that of water-treated *C. septempunctata*. However, chlorpyrifos, profenofos, and lambda-cyhalothrin had significantly greater effects on R_0_ and *T*, compared to the other insecticides (Table 2). Adult pre-oviposition period (APOP) (F = 51.20; *p* = 0.001) and total pre-oviposition period (TPOP) (F = 98.27; *p* = 0.002) were significantly prolonged in beetles treated with the six insecticides compared to those of water-treated beetles. Chlorpyrifos, profenofos, and lambda-cyhalothrin had greater effects on TPOP and APOP compared to those of the other three insecticides. Female fecundity was also reduced in beetles treated with insecticides compared to that of the control (water-treated) beetles (F = 698.27; *p* < 0.0001) (Table 2). A higher reduction in eggs/female was recorded in beetles treated with sublethal doses of profenofos, chlorpyrifos, and lambda-cyhalothrin than that from the other insecticides. Oviposition durations of females were significantly higher in water-treated beetles, compared to insecticide-treated beetles (F = 173.27; *p* < 0.0001).

**TABLE 2:** Population parameters (means ± SE) of *Coccinella septempunctata* F1 generation adults in response to sublethal exposure (LC_30_) to six insecticides.

Basic statistic	Control	Imidacloprid	Thiamethoxam	Lambda-cyhalothrin	Cypermethrin	Chlorpyrifos	Profenofos
Intrinsic rate of increase (r) (d−1)	0.121 ± 0.005a	0.120 ± 0.007b	0.119 ± 0.006b	0.106 ± 0.006c	0.113 ± 0.006b	0.108 ± 0.006c	0.107 ± 0.006c
Finite rate (λ) (d−1)	1.128 ± 0.006a	1.126 ± 0.007ab	1.122 ± 0.007ab	1.112 ± 0.006c	1.120 ± 0.006b	1.114 ± 0.007c	1.114 ± 0.007c
Net reproduction rate (R0)	293.1 ± 45.69a	248.99 ± 64.96b	234.90 ± 58.53b	191.03 ± 49.17d	204.28 ± 50.42c	210.97 ± 55.34c	224.05 ± 58.76c
Mean generation time (T) (d)	45.86 ± 0.84c	47.80 ± 0.80b	47.94 ± 1.15b	49.17 ± 0.88a	48.66 ± 0.83ab	49.44 ± 0.60a	49.66 ± 0.63a
APOP (d)	9.50 ± 0.31d	10.49 ± 0.30bc	9.91 ± 0.79c	12.0 ± 0.52a	10.76 ± 0.43b	11.90 ± 0.28a	11.72 ± 0.32a
TPOP (d)	34.30 ± 0.82d	36.30 ± 0.80b	36.16 ± 1.03bc	37.58 ± 0.96a	35.92 ± 0.79c	37.45 ± 0.66a	37.72 ± 0.67a
Fecundity	879.3 ± 18.61a	746.9 ± 30.77b	641.09 ± 41.73c	454.54 ± 69.73e	452.50 ± 70.46e	545.08 ± 74.66d	579.25 ± 79.66d
Oviposition duration (d)	35.80 ± 1.30a	31.59 ± 1.01b	28.0 ± 1.61c	24.50 ± 2.85d	24.38 ± 3.16d	26.91 ± 2.58cd	28.60 ± 2.72bc

Means sharing same letters in rows are not significantly different from one another at *p* > 0.05. Adult Pre-Oviposition Period (APOP) of female is the interval from adult emergence to first oviposition and the Total Pre-Oviposition Period (TPOP) of female is the duration from egg to first oviposition.

**FIGURE 2:**
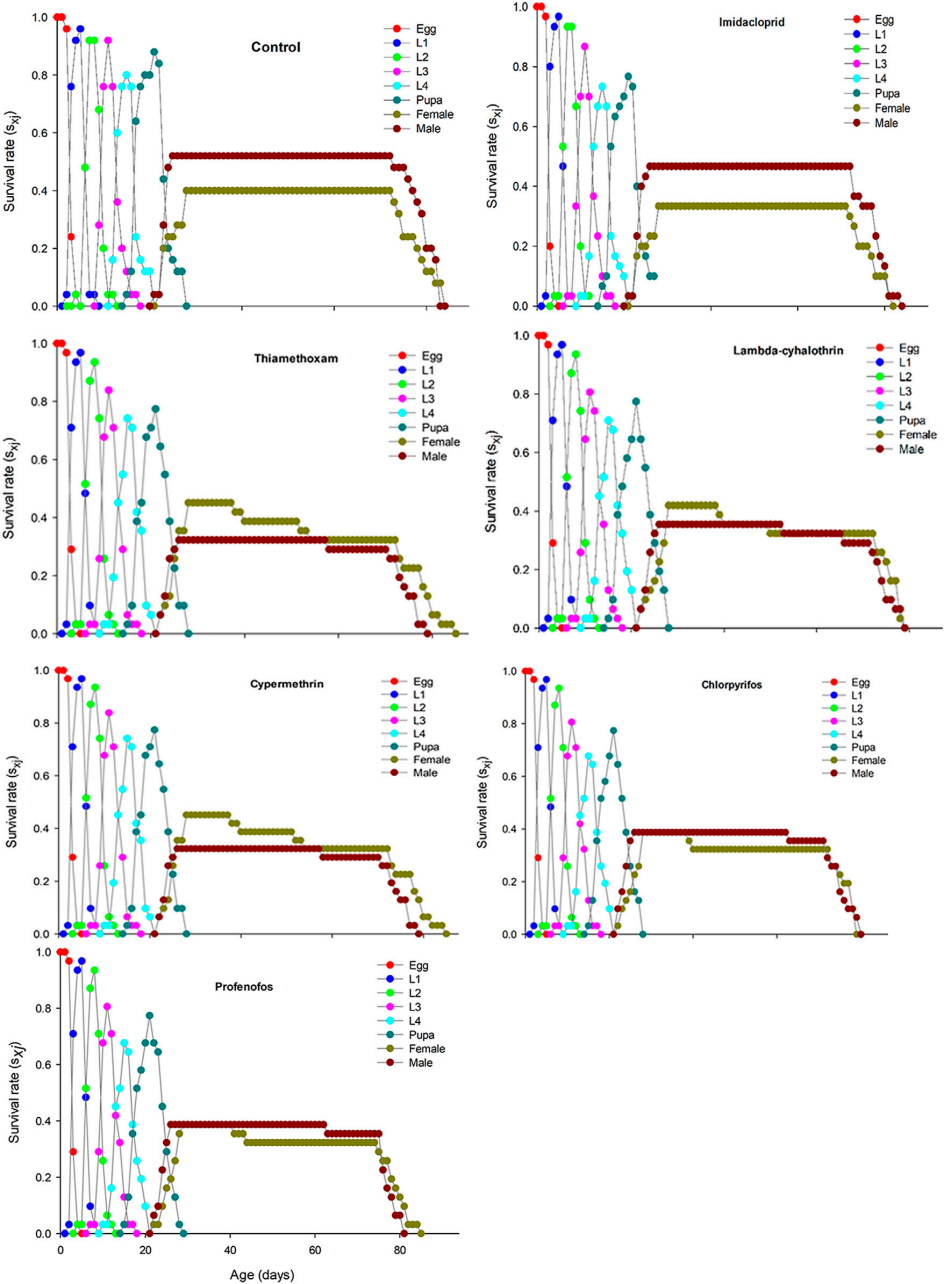
Age-stage specific survival (*sxj*) of *Coccinella septempunctata* (F_1_) treated with water (control) and sublethal concentrations (LC_30_) of synthetic insecticides.

**FIGURE 3:**
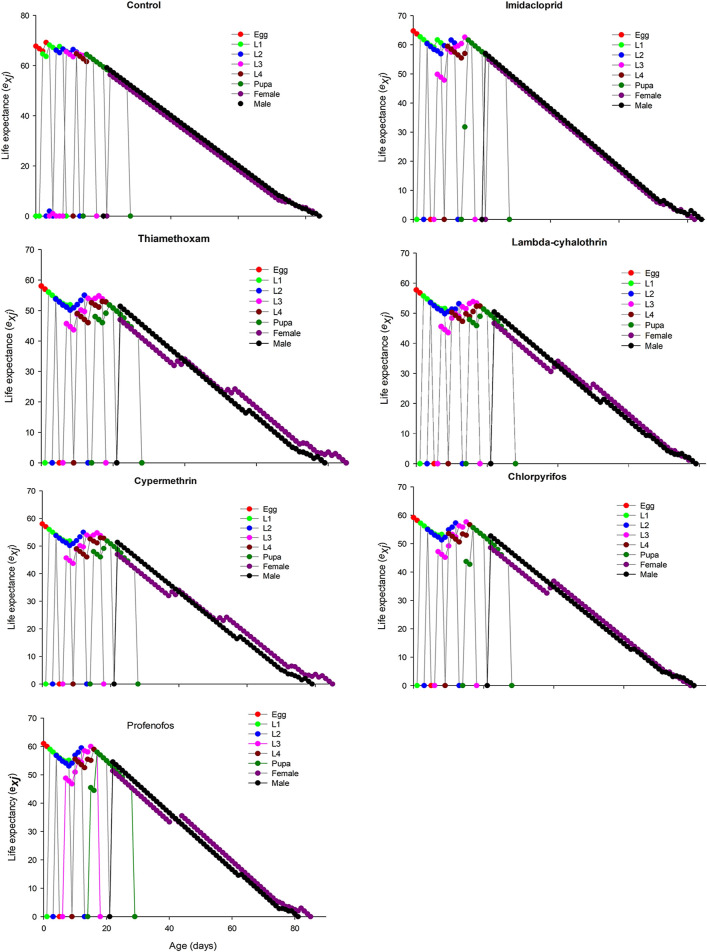
Age-stage specific life expectancy (e*xj*) of *Coccinella septempunctata* (F_1_) treated with water (control) and sublethal concentration (LC_30_) of synthetic insecticides.

**FIGURE 4:**
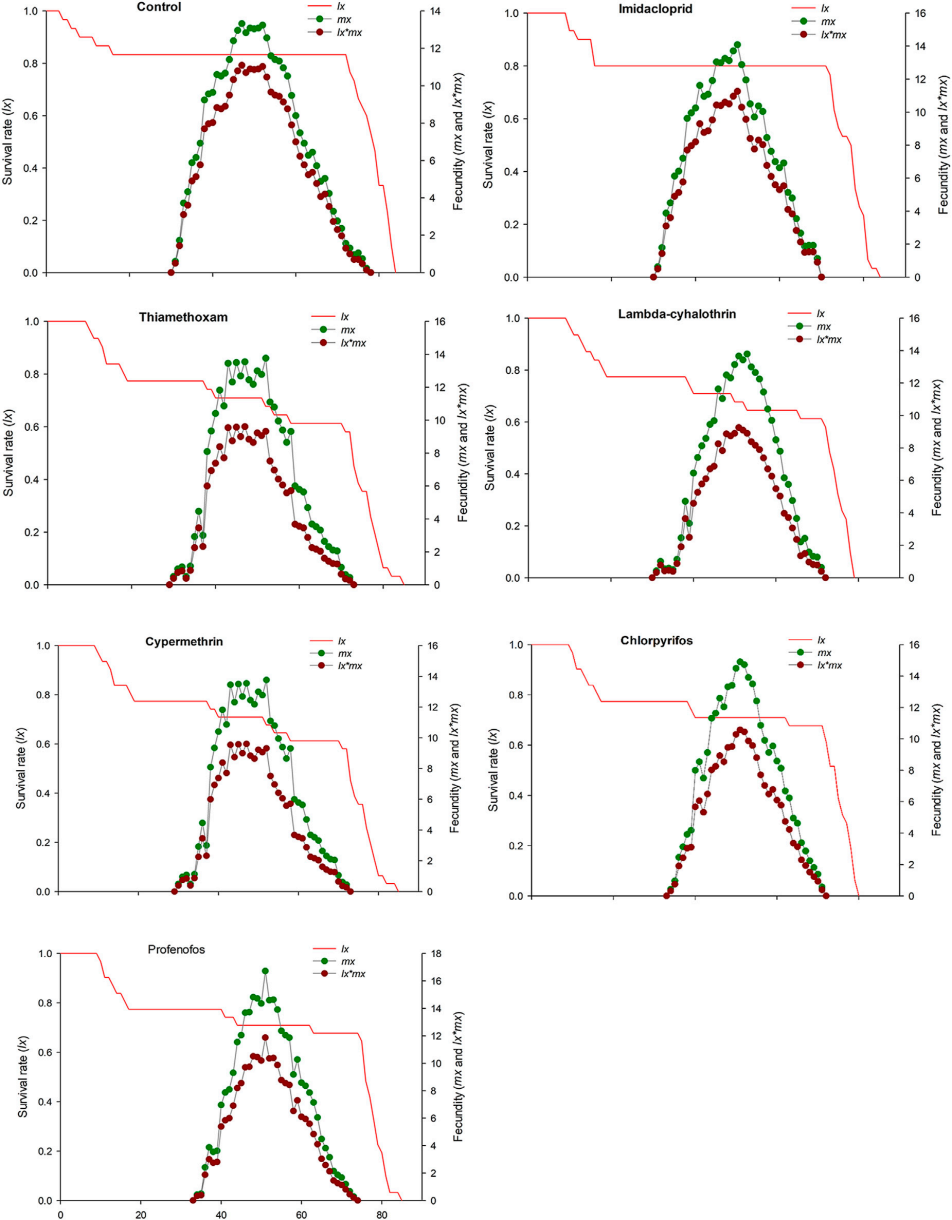
Age-stage specific survival rate (*lx*), age-specific fecundity (*mx*) and net maternity (*lx × mx*) of *C. septempunctata* (F_1_) treated with water (control) and sublethal concentration (LC_30_) of synthetic insecticides.

**FIGURE 5:**
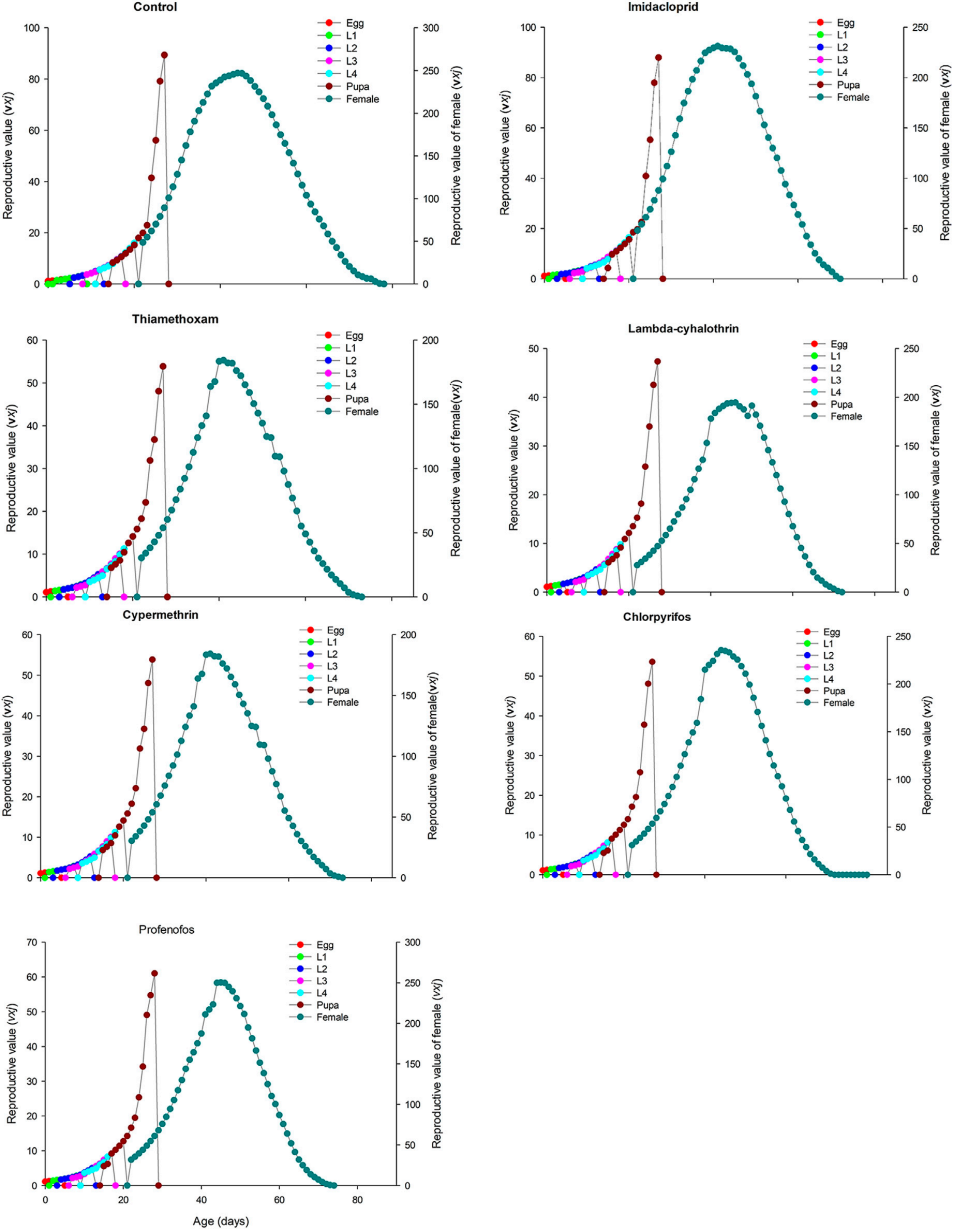
Age-stage specific reproductive value (*vxj*) of *Coccinella septempunctata* (F_1_) treated with water (control) and sublethal concentration (LC_30_) of synthetic insecticides.

The age-stage specific survival rate (sxj) curves showed the variation in different developmental stages of the coccinellids. There were distinct overlaps between the control and insecticidal treatments (Figure 2). The survival rate of adult males and females was decreased after treatment with lambda-cyhalothrin and chlorpyrifos, compared to the control and that of the other insecticidal treatments. The larval duration after treatments with lambda-cyhalothrin, profenofos, and chlorpyrifos was significantly reduced compared to that of control and other insecticidal treatments. The exj was significantly reduced in beetles treated with sublethal doses of all insecticides except imidacloprid, compared to that of the control treatment (Figure 3). The lx, mx, and lxmx are presented in Figure 4. Age-stage specific survival rate was significantly reduced in beetles treated with chlorpyrifos and lambda-cyhalothrin compared to that of beetles treated with water and other insecticides. The maximal survival time for control treatment was 82 days, which was higher than that under insecticidal treatments. Similarly, compared to control treatment, the vxj of *C. septempunctata* was significantly reduced after treatment with sublethal doses of all insecticides except for imidacloprid and chlorpyrifos (Figure 5). A higher reduction in vxj was observed in beetles treated with a sublethal dose of lambda-cyhalothrin followed by thiamethoxam compared to that from the other insecticides.

### 3.4 *C. septempunctata* functional response after feeding on insecticide-treated aphids

**FIGURE 6:**
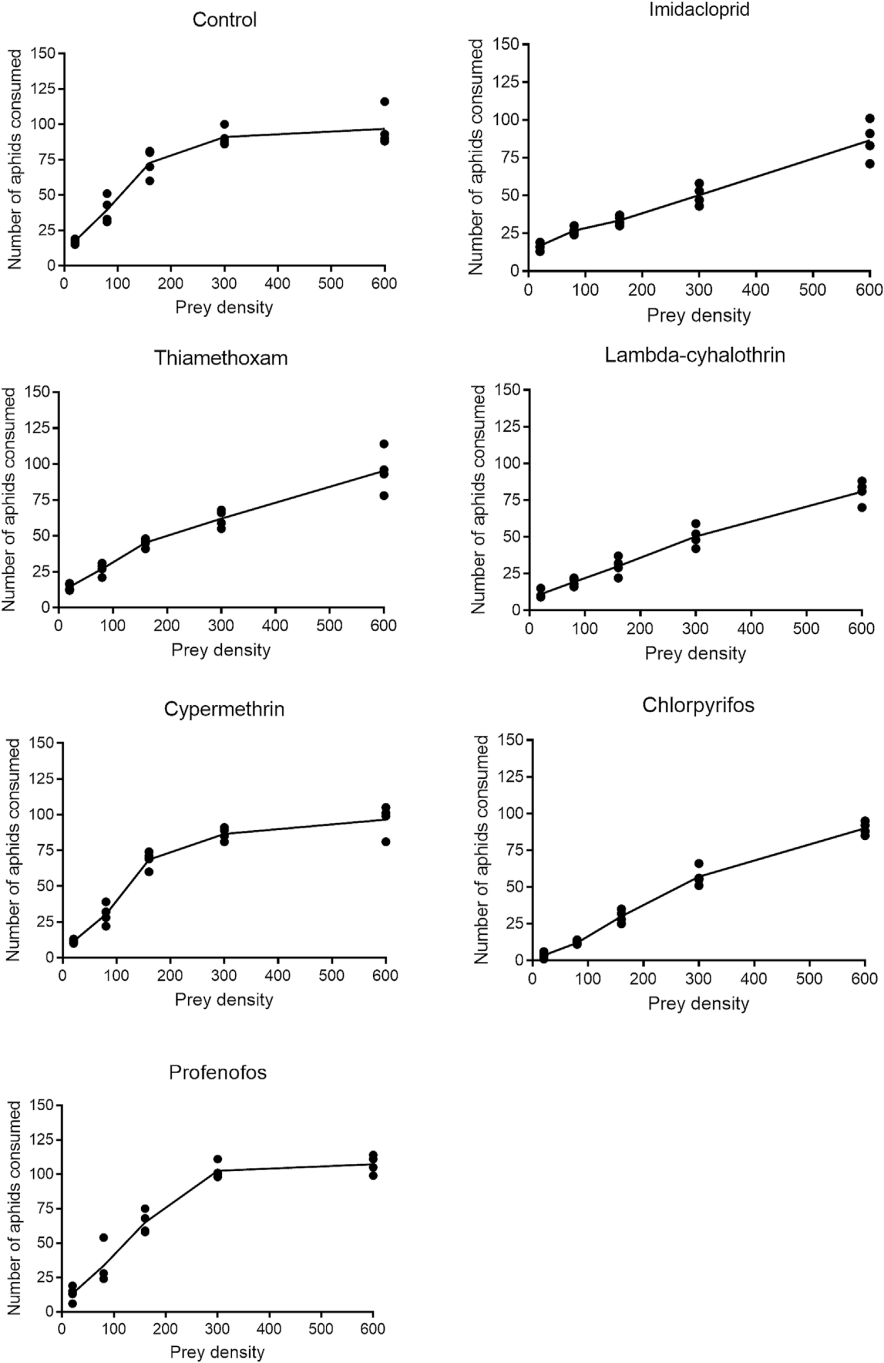
Functional responses of *Coccinella septempunctata* adults exposed to sublethal doses of synthetic insecticides and control.

The functional response of F_1_ coccinellid adults to B. brassicae aphids fitted o the Holling’s type II curves (Figure 6). Logistic regression analysis coefficients of the proportion of sublethal (LC_30_) dose-treated aphids devoured by adult coccinellids are presented in Supplementary Table S2. Values of the parameters α, Th and T/Th derived from all treatments are presented in Table 3. The instantaneous attacking rate (α) derived from control treatment remained significantly higher (0.075) compared to that from insecticidal treatments. Significantly minimum attack rate was observed in beetles which fed on aphids treated with lambda-cyhalothrin (0.001), followed by chlorpyrifos (0.011) and thiamethoxam (0.010). The maximum handling time (Th) (0.069) was recorded for beetles which fed on aphids treated with chlorpyrifos, followed by lambda-cyhalothrin (0.051) and profenofos (0.050). The handling time of prey by beetles treated with imidacloprid, thiamethoxam and cypermethrin did not differ significantly. The minimum handling (0.017) time was recorded under control treatment.

**TABLE 3:** Parameters for functional response model for adult *Coccinella septempunctata* exposed to sublethal concentrations (LC_30_) of six insecticides.

Treatment	A	Th	T/Th	R2
Control	0.075a	0.017d	135.5	0.98
Imidacloprid	0.020c	0.033c	72.72	0.96
Thiamethoxam	0.010cd	0.038c	63.15	0.92
Profenofos	0.021c	0.050b	82.75	0.89
Chlorpyriphos	0.011cd	0.069a	48.01	0.96
Lambda-cyhalothrin	0.001e	0.051b	77.41	0.99
Cypermethrin	0.031bc	0.044c	71.56	0.85

a–attack rate; Th–handling time; T/Th, maximum attack rate; Means sharing same letters in rows are not significantly different from one another at *p* > 0.05.

## 4 Discussion

Insecticides are mainly used as a foliar spray on canola and other winter crops. Therefore, predators of insect pests, including coccinellid beetles are likely to be exposed to insecticides while foraging on the treated plants and pests. Exposure to pesticides may cause various adverse effects on the life-history traits and feeding behaviour of coccinellid beetles. In the current study, the sublethal effect of six insecticides belonging to three chemical classes was presented with experimental evidences. We found that the sublethal doses (LC_30_) of the six insecticides adversely affected the biological parameters of *C. septempunctata* (F_0_ generation). Lambda-cyhalothrin and chlorpyrifos increased the pupal duration compared to that from the other insecticides. All the tested insecticides reduced the adult emergence, fecundity rates, weight of adults, and survival compared to the control treatment. These results are similar to those from other studies which showed that most of the tested insecticides impaired the reproduction and survival of *C. septempunctata* and other predators (Yu et al., 2014; Skouras et al., 2017; Kang et al., 2018; Jiang et al., 2019; Noelia et al., 2022).

Transgenerational impacts of the six insecticides on the predatory beetle, *C. septempunctata*, were studied by age-stage, two-sex life table model analyses. Results showed that the second, third, fourth instar and pupation stages of *C. septempunctata* in the F_1_ generation were prolonged when their parental generations (F_0_) were exposed to sublethal doses of all insecticides. This prolongation in the larval and pupal development may be due to physiological effects such as reduced food assimilation or stunted development at the cost of detoxification mechanism (Skouras et al., 2017). However, treatment with a sublethal dose of lambda-cyhalothrin had higher significant effects on the second instar than the other insecticides. Similarly, the sublethal dose of lambda-cyhalothrin, chlorpyrifos and profenofos prolonged the third instar duration compared to the other three insecticides. All insecticides reduced the adult duration compared to the control treatment, and beetles treated with a sublethal dose of lambda-cyhalothrin had the least adult duration. The total longevity of beetles treated with the sublethal dose was significantly reduced compared to that under control treatment. The differences between the sublethal effects of insecticides on insect depend on the variations in the structure and efficacy of insecticides (Alkassab and Kirchner 2016; Skouras et al., 2017; Dai et al., 2019; Tan et al., 2021).

The sublethal doses of all insecticides adversely influenced the reproductive performance of the F_1_ generation. Reductions in the fecundity of females exposed to insecticides may result from both physiological and behavioural effects (Desneux et al., 2007; Wu et al., 2022). The duration of the F1 generation’s pre-oviposition period (APOP and TPOP) and the oviposition period changed significantly in beetles treated with lambda-cyhalothrin, chlorpyrifos, and profenofos (Desneux et al., 2004; De Castro et al., 2013). Moreover, there was a significant reduction in the number of offspring (F_2_) from parent individuals exposed to sublethal doses of all insecticides (Fernandes et al., 2010). These insecticides might have disrupted the very precise coordination of the nervous and hormonal systems in the insects, resulting in a breakdown in behavioural and physiological events related to oviposition (Desneux et al., 2007).

Population parameters such as intrinsic (*r*) and finite (*λ*) rates of increase, net reproductive rate (*R*_0_) and mean generation time (*T*) can be useful to better understand the population dynamics of insects (Liu et al., 2017; Zheng et al., 2017; Negi et al., 2018; Jiang et al., 2019). Results showed that the treatment of F_0_ with sublethal doses of all insecticides significantly reduced the *r, λ,* and *R*_0_ parameters of the F1 generation.

However, the sublethal doses of lambda-cyhalothrin, chlorpyrifos, and profenofos significantly reduced *r* and *λ* than sublethal doses of imidacloprid and thiamethoxam. In general, the fluctuations in demographic features demonstrated that sublethal concentrations pyrethroid and organophosphate insecticides had the greatest effects on the reproduction and survival of *C. septempunctata* transgenerationally than the two neonicotinoids.

Functional response is generally determined to assess the feeding efficiency of predatory species to agricultural pests (Juliano 2001; Xue et al., 2009; He et al., 2012; Sarkar et al., 2022). Sublethal exposure to all insecticides impaired the functional responses and prolonged the handling time of aphid by the predator beetle. This agrees with studies of He et al. (2012) and Yao et al. (2015) which showed that thiamethoxam and imidacloprid prolonged the handling time of *Bemisia tabaci* eggs by the predatory coccinellid beetle, *Serangium japonicum* at sublethal doses. However, a maximum handling time was observed when beetles were offered aphids treated with chlorpyrifos, followed by profenofos (GholamzadehChitgar et al., 2014).

Overall, sublethal doses of all the tested insecticides impaired the fitness and predation efficacy of *C. septempunctata* individuals up to two successive generations. However, the effects of the pyrethroids and organophosphates were greater, compared to neonicotinoids. This agrees with previous studies (Ahmad et al., 2011). Organophosphates and pyrethroids have low selectivity to insect predators and parasitoids than neonicotinoids (Galvan et al., 2002; Fernandes et al., 2010). This greater negative effects of the two insecticide groups, may be associated with their pro-insecticide activities. Moreover, organophosphates and pyrethroids have lipophilic characteristic and can be adsorbed in the beetle cuticle very easily when topically applied (GholamzadehChitgar et al., 2014; Costa 2015). This may be responsible for thegreater negative effects they have on insects.

## 5 Conclusion

Our laboratory experiments demonstrated that all the tested insecticides had adverse effects on the fitness and predation efficacy of *C. septempunctata* individuals. However, among these, the organophosphates and pyrethroids had greater negative effects on the population parameters of beetles compared to the neonicotinoids. Therefore, these neonicotinoids have some potential which can enable their integration into IPM programs. Additionally, we also evaluated the demographic and transgenerational effects mediated by exposure to sublethal doses of these insecticides. Future work will focus on the mechanisms underlying these adverse effects of the sublethal doses of the insecticides on *C. septempunctata*.

## Data Availability

The original contributions presented in the study are included in the article/Supplementary Material. Further inquiries can be directed to the corresponding authors.
