# Supplementary material for: Sublethal and transgenerational effects of synthetic insecticides on the biological parameters and functional response of Coccinella septempunctata (Coleoptera: Coccinellidae) under laboratory conditions
Source: Front Physiol. 2023 Jan 16;14:1088712. doi: 10.3389/fphys.2023.1088712 (PMC9885102; doi:10.3389/fphys.2023.1088712)
Supplement: Supplementary file 1 [file Table1.DOCX]

**Supplementary Information**

**Sublethal and transgenerational effects of synthetic insecticides on the biological parameters and functional response of *Coccinella septempunctata* (Coleoptera, Coccinellidae) under laboratory conditions**

**Table S1**

List of selected synthetic insecticides bioassayed against *Coccinella septempunctata* in the study.

| Sr. # | Common name | Trade name & formulation | Company name | Chemical group | Mode of action |
| --- | --- | --- | --- | --- | --- |
| 1 | Imidacloprid | Confidor 200 SL | Bayer Crop Science Pakistan. | Neonicotinoids | Competitive modulators of nicotinic acetylcholine receptor (nAChR) and mimic the agonist action of acetylcholine |
| 2 | Thiamethoxam | Actara 25 WG | Syngenta Pakistan LTD |  |  |
| 3 | Profenofos | Curacron 50 EC | Syngenta Pakistan LTD | Organophosphates | Inhibit acetylcholinesterase, causing hyperexcitation |
| 4 | Chlorpyrifos | Lorsban 40 EC | Syngenta Pakistan LTD |  |  |
| 5 | Lambda-cyhalothrin | Karate 50 EC | Syngenta Pakistan LTD | Pyrethroids | Sodium channel modulators, keep sodium channels open, causing hyper-excitation and, in some cases block nerves |
| 6 | Cypermethrin | Arrivo 10 EC | FMC Pakistan LTD |  |  |

**AGE-STAGE, TWO-SEX LIFE TABLE**

Net reproductive rate (R0), age-specific survival rate (lx) and age-specific fecundity (mx) were calculated using the following equations;

$$R_{0}=\sum_{x=0}^{\infty} l_{x}m_{x}$$

$$l_{x}=\sum_{j=1}^{k} s_{xj}$$

$$m_{x}=\frac{\sum_{j=1}^{k} s_{xj}f_{xj}}{\sum_{j=1}^{k} s_{xj}}$$

The intrinsic rate of increase (*r*) was calculated using

$$r= \sum_{x=0}^{\infty} ⅇ^{-r\left( x+1 \right)}l_{x}m_{x}$$

The finite rate was calculated as

$$\lambda=ⅇ^{r}$$

The mean generation time “the length of time that a population needs to increase to *R*_0_-fold of its population size at the stable age-stage distribution” was calculated using following equation:

$$T=lnRo/r$$

The life expectancy (*e_xj_*) “the length of time that an individual of age *x* and stage *j* is expected to live” was calculated according to (Chi and Su 2006)

$$e_{xj}=\sum_{i=x}^{\infty} \sum_{y=j}^{\beta} S_{iy}^{'}$$

The reproductive value (*v_xj_*) was calculated using following equation

$$V_{xj}=\frac{ⅇ^{r\left( x+1 \right)}}{s_{xj}}\sum_{i=x}^{\infty} ⅇ^{-r\left( i+1 \right)}\sum_{y=j}^{\beta} S_{iy}^{'}f_{iy}$$

**Functional Response**

Logistic regression was used to determine functional response as a proportion of aphids eaten as a function of initial prey density, and the random predator equation was fitted for the determination of the type of functional response. The logistic regression model was used to determine the shape by using the proportion of prey eaten (Na/ No) as a function of preys offered (No) (Juliano, 2001). Hence the following polynomial equation was fitted on the data

$$\frac{N_{e}}{N_{0}}=\frac{\exp(P_{0}+P_{1}N_{0}+P_{2}N_{0}^{2}+P_{3}N_{0}^{3})}{1+\exp(P_{0}+P_{1}N_{0}+P_{2}N_{0}^{2}+P_{3}N_{0}^{3})}$$

Ne is the number of prey eaten, N0 is the initial number of prey, and P_0_, P_1_, P_2_, and P_3_ are the intercept, linear, quadratic, and cubic coefficients, respectively

If the negative relationship between the increased prey density and prey consumption rate occurred (linear parameter significant and negative), the type II functional response was selected for those data. If the linear parameter were significant and positive, increased prey density would result in a positive predation response, and the type III functional response model was chosen for that data set. The coefficients of polynomial logistic regression were determined using the function" glm" in R software. These parameters were estimated using the CAT MOD procedure. After the determination of the type of functional response (Table S2), the data i.e., the number of aphids preyed upon by different stages of coccinellids at different densities were analyzed by fitting Rogers' Type II Random Predator Equation ([Rogers, 1972](#_ENREF_25)) with the help of non-linear least square regression to determine the parameters of Type II functional response.

Rogers type II Random Predator Equation is given by

$$Na = No \{1- exp [a (Th No - T)]\}$$

Na is the number of preys eaten, No is the number of preys offered; a is attack rate, Th is handling time, T is a time of confinement (24 hours). T is determined by the coefficients of attack rate and handling time using non-linear least square regression as suggested by ([Rogers, 1972](#_ENREF_25)) the function "nls" provided by the R-software was used**.**

**Table S2**

Coefficients of the logistic regression analysis of the proportion of LC_30_ treated aphids eaten by *Coccinella septempunctata.*

|  | Type | Intercept (P_0_) | Linear (P_1_) | Quadratic (P_2_) | Cubic (P_3_) |
| --- | --- | --- | --- | --- | --- |
| Control | II | -0.59 ± 0.04 | -3.13 ± 0.20 | 1.21 ± 0.23 | -0.80 ± 0.22 |
| Imidacloprid | II | -0.94 ± 0.05 | -2.96 ± 0.22 | 1.69 ± 0.24 | -0.81 ± 0.24 |
| Thiamethoxam | II | -0.91 ± 0.04 | -3.07 ± 0.23 | 2.27 ± 0.25 | -1.35 ± 0.25 |
| Profenofos | II | -1.32 ± 0.05 | -2.25 ± 0.21 | 1.65 ± 0.25 | -0.73 ± 0.27 |
| Chlorpyrifos | II | -1.32 ± 0.05 | -2.25 ± 0.23 | 1.65 ± 0.25 | -0.73 ± 0.25 |
| Lambda-cyhalothrin | II | -1.52 ± 0.06 | -1.15 ± 0.26 | 0.20 ± 0.27 | -0.002 ± 0.26 |
| Cypermethrin | II | -1.90 ± 0.07 | -1.07 ± 0.28 | 0.86 ± 0.30 | -0.30 ± 0.30 |
