# Supplementary figures and images for: Sublethal and transgenerational effects of synthetic insecticides on the biological parameters and functional response of Coccinella septempunctata (Coleoptera: Coccinellidae) under laboratory conditions
Source: Front Physiol. 2023 Jan 16;14:1088712. doi: 10.3389/fphys.2023.1088712 (PMC9885102; doi:10.3389/fphys.2023.1088712)

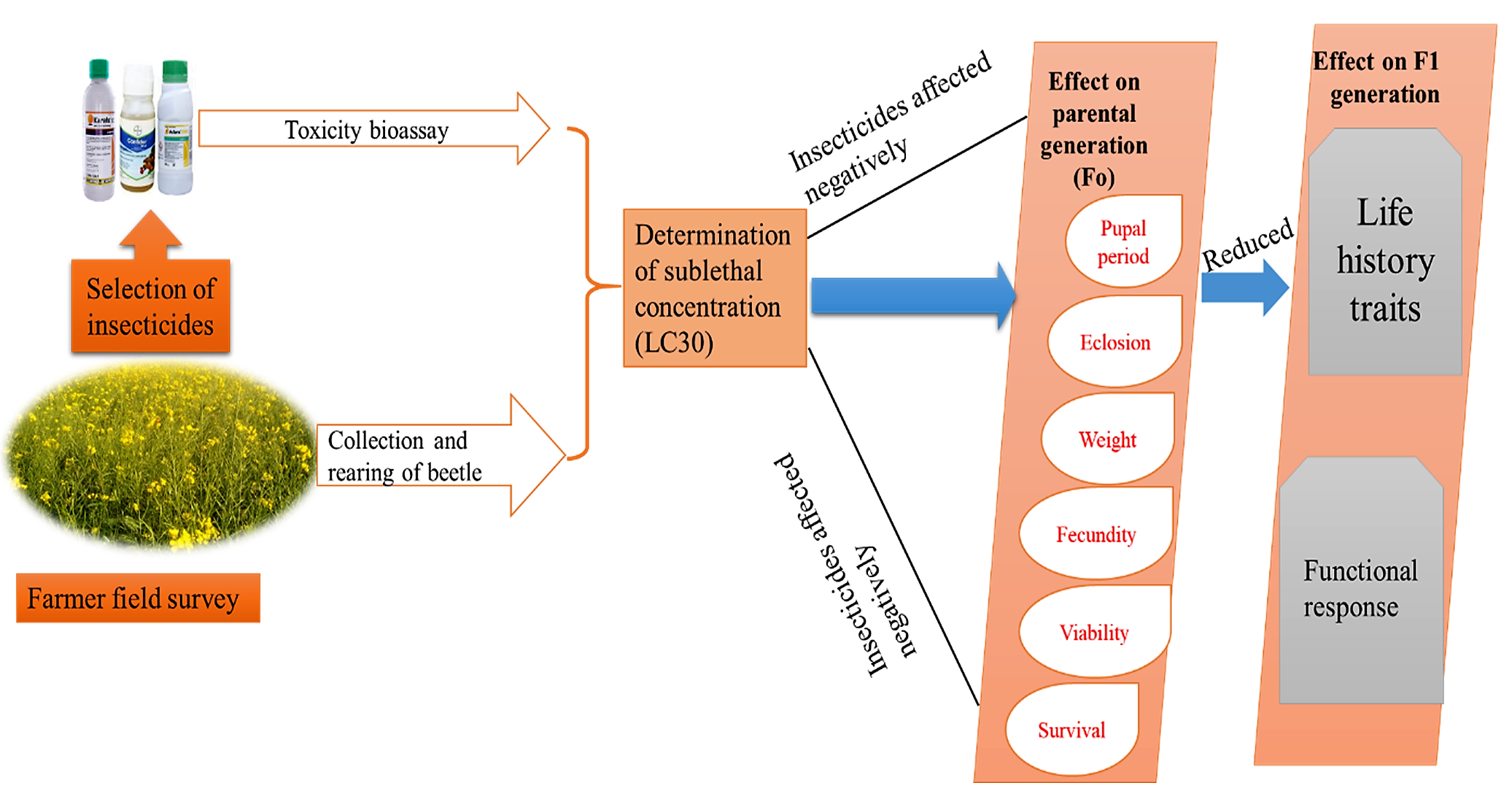

Supplement: Supplementary file 2 [file Image1.TIF]
